# SEOM-GEINO clinical guideline of systemic therapy and management of brain central nervous system metastases (2021)

**DOI:** 10.1007/s12094-022-02803-0

**Published:** 2022-03-08

**Authors:** María Martínez-García, Sonia Servitja Tormo, Noelia Vilariño Quintela, Ana Arance Fernández, Alfonso Berrocal Jaime, Blanca Cantos Sánchez de Ibargüen, Sonia Del Barco Berrón, Rosario García Campelo, Regina Gironés Sarrió, Juan Manuel Sepúlveda-Sánchez

**Affiliations:** 1 Medical Oncology Department, Hospital del Mar, Barcelona, Spain; 2 CIOCC HM Delfos, Barcelona, Spain; 3 Medical Oncology Department, Institut Català d’Oncologia L’Hospitalet, L’Hospitalet de Llobregat, Barcelona, Spain; 4 Medical Oncology Department, Hospital Clínic, Barcelona, Spain; 5 Medical Oncology Department, Consorcio Hospital General Universitario de Valencia, Valencia, Spain; 6 Medical Oncology Department, Hospital Universitario Puerta de Hierro Majadahonda, Madrid, Spain; 7 Medical Oncology Department, Hospital Universitari Dr. Josep Trueta, ICO, Girona, Spain; 8 Medical Oncology Department, Complexo Hospitalario Universitario A Coruña (CHUAC), A Coruña, Spain; 9 Medical Oncology Department, Hospital Universitari i Politècnic la Fe, Valencia, Spain; 10 Medical Oncology Department, Hospital Universitario, 12 de Octubre, Madrid, Spain

**Keywords:** Brain metastasis, Blood–brain barrier, Targeted therapies, Immunotherapy, Guidelines

## Abstract

Central nervous system (CNS) dissemination is a severe complication in cancer and a leading cause of cancer-related mortality. Brain metastases (BMs) are the most common types of malignant intracranial tumors and are reported in approximately 25% of patients with metastatic cancers. The recent increase in incidence of BMs is due to several factors including better diagnostic assessments and the development of improved systemic therapies that have lower activity on the CNS. However, newer systemic therapies are being developed that can cross the blood–brain barrier giving us additional tools to treat BMs. The guidelines presented here focus on the efficacy of new targeted systemic therapies and immunotherapies on CNS BMs from breast, melanoma, and lung cancers.

## Introduction

Central nervous system (CNS) dissemination is a severe complication in cancer. Brain metastases (BMs) are reported in approximately 25% of patients with metastatic cancer [1] and its incidence has been increasing mainly due to the impact of systemic therapies on survival and the improvement and availability of radiological techniques and screening. Lung cancer is the most frequent primary tumor for BMs, followed by breast cancer and melanoma. Globally, there is a 40–60% risk of presenting BM in melanoma, 20–45% in lung cancer, and 5–30% in breast cancer [1]. Selection of the systemic therapy should be determined not only by the histological tumor type but also by the specific molecular subtype since both of these factors influence risk. Clinical practice guidelines for diagnosis, treatment (including surgery and radiotherapy), and follow-up of patients with BM have been published recently [2, 3]. The present guidelines from the Spanish Society of Medical Oncology (SEOM) have been developed with the consensus of 10 medical oncologists from SEOM and the Spanish Group of Neuro Oncology (GEINO). These guidelines will focus on systemic management of BM from breast, melanoma, and lung cancer, as well as recommendations for treatment of elderly patients.

## Methodology

Studies published in peer-reviewed journals were reviewed for the SEOM-GEINO Guidelines. The US Agency for Healthcare Research and Quality Service Grading System (USPSTF) was used to assign levels of evidence and grades of recommendation [4].

## Management of symptoms in BM

Brain edema is a common condition found in the magnetic resonance Imaging (MRI) and computed tomography (CT) scans of patients with BMs but the need for anti-edema treatment is primarily based on a patient´s symptoms and not only on radiological findings. Dexamethasone is the most frequently used steroid for this purpose with standard doses between 4 and 16 mg/day [5].

Seizures are a major issue in brain tumor patients but, unfortunately, there are no randomized trials assessing the efficacy of antiepileptic drugs (AEDs) in this population. However, levetiracetam may be the most appropriate drug since it has significant efficacy in seizure control and has no significant drug interactions [6]. Newer drugs, such as lacosamide and brivaracetam, can be also recommended since both have no reported drug interactions and their adverse effects are manageable [7, 8]. There is insufficient evidence to recommend prophylaxis with AEDs [9]. Venous thromboembolism is significantly increased in BM patients but anti-coagulants can increase the risk of intracranial hemorrhage. Therefore, the prescription of prophylactic anticoagulation in these patients requires a careful risk–benefit assessment.

## Management of breast cancer BM

### Management of HER2-positive breast cancer BMs

**Fig. 1:**
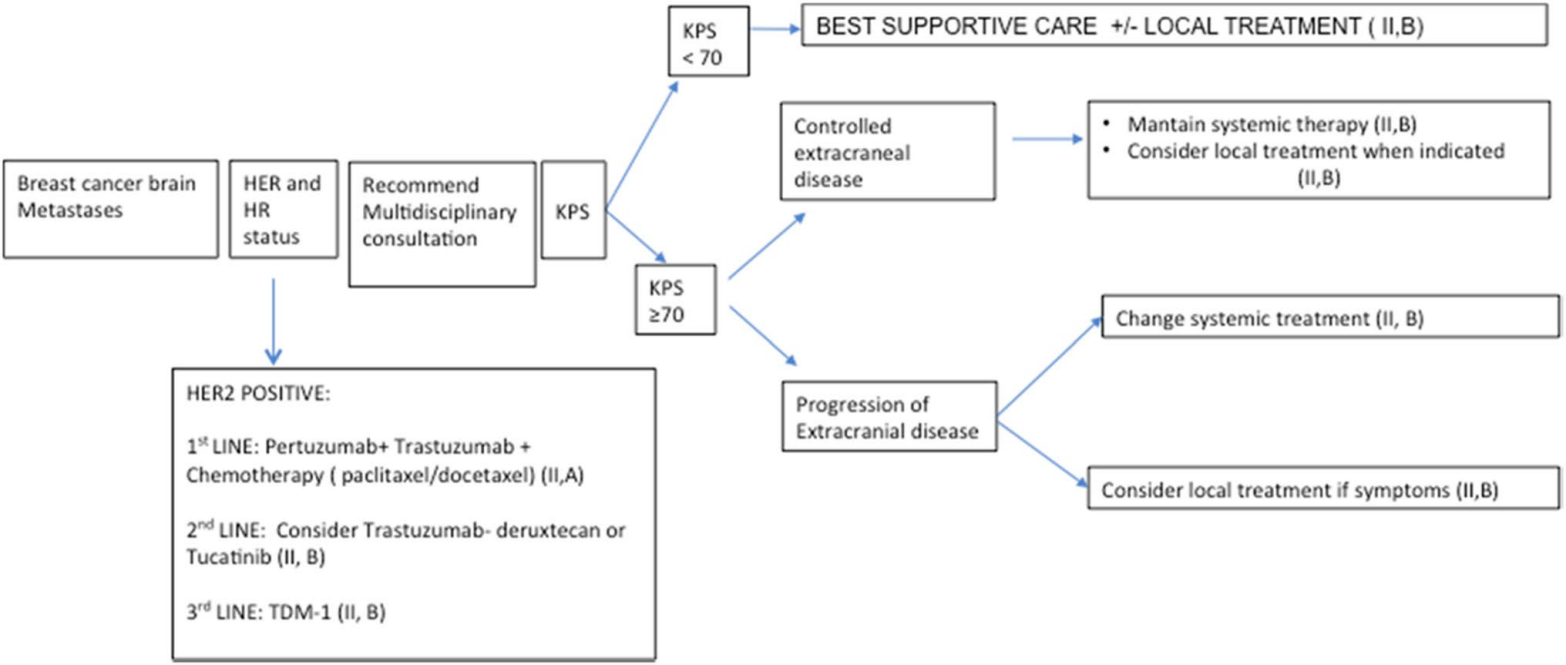
Breast cancer BM algorithm

BM is the leading cause of death in patients with HER2-positive breast cancer, despite approved anti-HER2 treatment options [10]. Promising new molecules have demonstrated activity in heavily pretreated HER2-positive metastatic breast cancer patients. In the phase III HER2CLIMB trial [11], tucatinib, an oral tyrosine kinase inhibitor (TKI) with high selectivity for HER2, was combined with trastuzumab and capecitabine in 612 patients with previously treated HER2-positive metastatic breast cancer. Among the 219 patients with BM, progression-free survival (PFS) at 1 year was 24.9% in the tucatinib combination group and 0% in the placebo combination group (HR 0.48; 95% CI 0.34–0.69; *p* < 0.001), and the median PFS for CNS-target lesions was 9.9 months (HR 0.32; 95% CI 0.22–0.48, *p* < 0.0001) compared to 4.2 months in the control arm. The overall response rate (ORR) was 47% vs 20% and prolonged overall survival (OS) was 18.1 vs 12 months (HR 0.58; CI 95% = 0.40–0.85; *p* = 0.005) in the tucatinib vs control arms, respectively (see Fig. 1).

In the NALA Trial [12], neratinib, another HER2-selective TKI, was compared against lapatinib, with both drugs in combination with capecitabine. Mean PFS at 24 months in 101 BM patients was 7.8 months with neratinib vs 5.5 months with lapatinib (HR 0.66), and mean OS through 48 months was 16.4 vs 15.4 months, respectively (HR 0.90). In patients with target CNS lesions at baseline (*n* = 32), confirmed intracranial objective response rates were 26.3% and 15.4%, respectively.

For the Destiny-Breast 01 [13] phase II study, trastuzumab deruxtecan, an antibody–drug conjugate, had demonstrated activity in patients pretreated with trastuzumab emtansine. Although only 13% of the included patients had BMs, this subgroup had a CNS response rate of 55%. In addition, only 8% of all patients showed brain progression.

### Management of BM for triple-negative breast cancer (TNBC)

The incidence of BM is as high as 46% among patients with advanced TNBC, with 14% presenting BM in the initial diagnosis of breast cancer [14]. BM occurs earlier and is more frequently accompanied by extracranial systemic lesions. In addition, it has a poor clinical prognosis, and is defined as refractory breast cancer due to its resistance to treatment. Patients with TNBC and BM also have a much shorter survival time, with a median OS of approximately 6 months and the worst breast cancer-specific survival and OS [15]. Furthermore, gene expression patterns in primary TNBC do not predict the occurrence of BM in this population [16].

To date, there is no standard treatment for BM-TNBC [17]. Only preliminary and non-randomized studies have been conducted on these patients using chemotherapy which have yielded varying results. Radiation therapy to the brain compromises the blood–brain barrier (BBB) and reduces expression of the efflux transporter P-glycoprotein (P-gp) [18]. P-gp effluxes a broad spectrum of natural compounds including chemotherapeutic drugs, such as anthracyclines, taxanes, and epipodophyllotoxin. However, carboplatin [19], 5-fluorouracil, and capecitabine [20] are capable of crossing the BBB and can be used to treat BM-TNBC without radiation therapy.

## General recommendations for breast cancer BM

Figure 1.

## Management of melanoma BM

### Targeted therapies

**Fig. 2:**
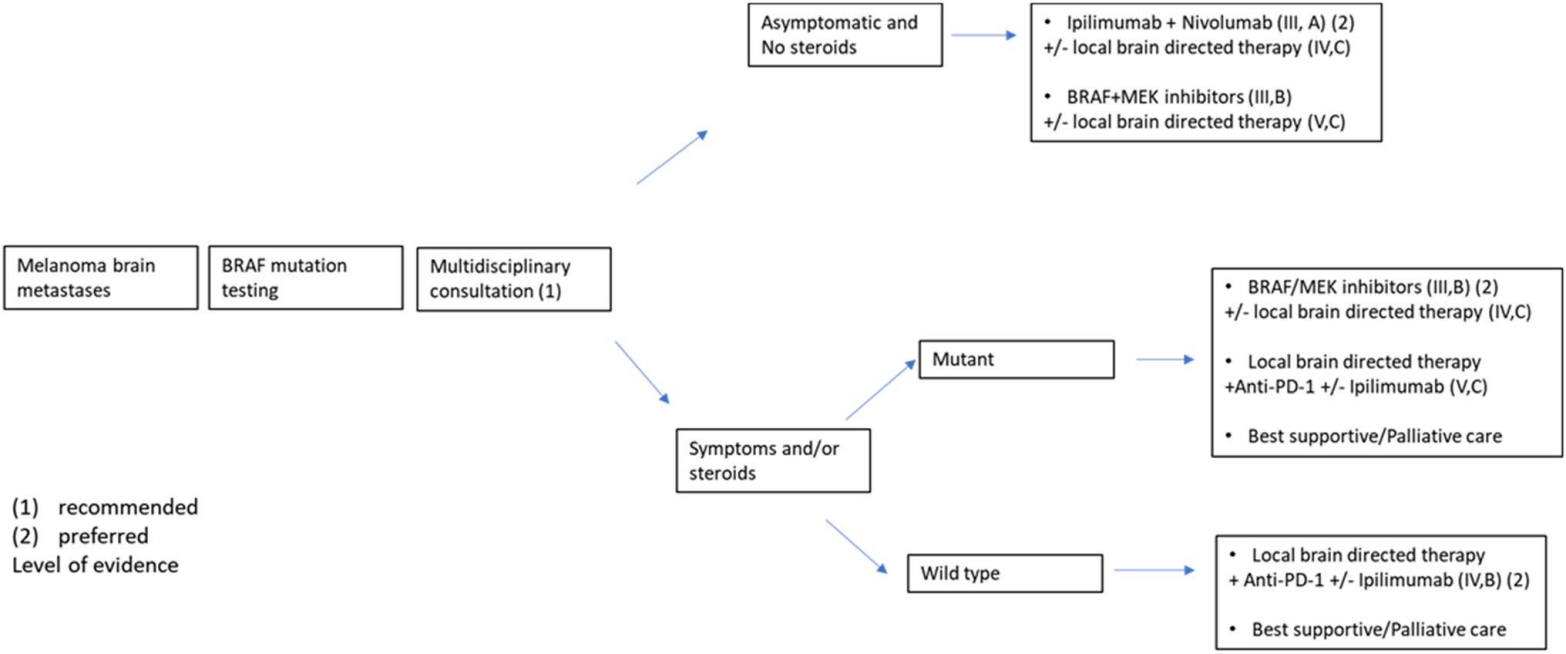
Melanoma BM algorithm

Among patients with BRAF-mutant melanoma and brain metastases (MBM), treatment with the BRAF inhibitors dabrafenib or vemurafenib leads to response rates of 20% and 38%, respectively, in patients with radiotherapy-naïve disease [21, 22]. Furthermore, the combination of dabrafenib with trametinib was evaluated in the COMBI-MB study [23]. Among the 125 patients enrolled, 76 BRAF^V600^ patients were asymptomatic MBM and with no previous local brain therapy, (cohort A); 16 BRAF^V600^ patients were asymptomatic MBM and with previous local brain therapy (cohort B); and 16 BRAF^V600D/K/R^ patients were asymptomatic MBM with or without previous local therapy (cohort C) and 17 BRAF ^V600D/K/R^ symptomatic melanoma brain metastases with or without previous local brain therapy, and an ECOG performance status of 0, 1, or 2 (cohort D). An intracranial response was achieved in 58%, 56%, 44%, and 59% of patients in cohorts A, B, C, and D, respectively. In cohort A, the median PFS was 5.6 months, the median OS was 10.8 months, and adverse events greater than grade 3 or 4 were reported in 48% of patients. Median PFS was almost half (5.6 vs 10.1 months) as compared to that observed with the same treatment in patients with extracranial disease [24], which suggests an earlier treatment failure in the brain. Triplet therapy including anti-PD-1/L1 is being explored in BRAF-mutant MBM to increase intracranial efficacy (NCT 03625141) (Fig. 2). 

### Immunotherapies

Systemic treatment with immunotherapy has improved the efficacy of MBM treatment. Monotherapy treatment with either anti-CTLA-4 or anti-PD-1 in BM patients reproduces the systemic efficacy observed in these patients. Intracranial response rates have been reported as 16% for anti-CTLA-4 and 20–26% for anti-PD-1 [25]. The activity of ipilimumab in combination with nivolumab was evaluated in two phase II studies, showing a high rate of durable intracranial responses (51–54%) in patients with asymptomatic MBM [26–28]. However, this scheme has shown limited efficacy in patients with symptomatic metastases or receiving steroid therapy [28]. Addition of localized treatment, such as surgery or radiotherapy, could increase brain control and survival [29]. Prospective randomized clinical trials are needed to better delineate the optimal associations of immunotherapy and radiotherapy [30]. At least one retrospective study has suggested that it is safe to interrupt treatment when a complete response is achieved.

## General recommendations for melanoma BM

Figure 2.

## Management of lung cancer BM

### EGFR-mutated non-small cell lung cancer (NSCLC)

**Fig. 3:**
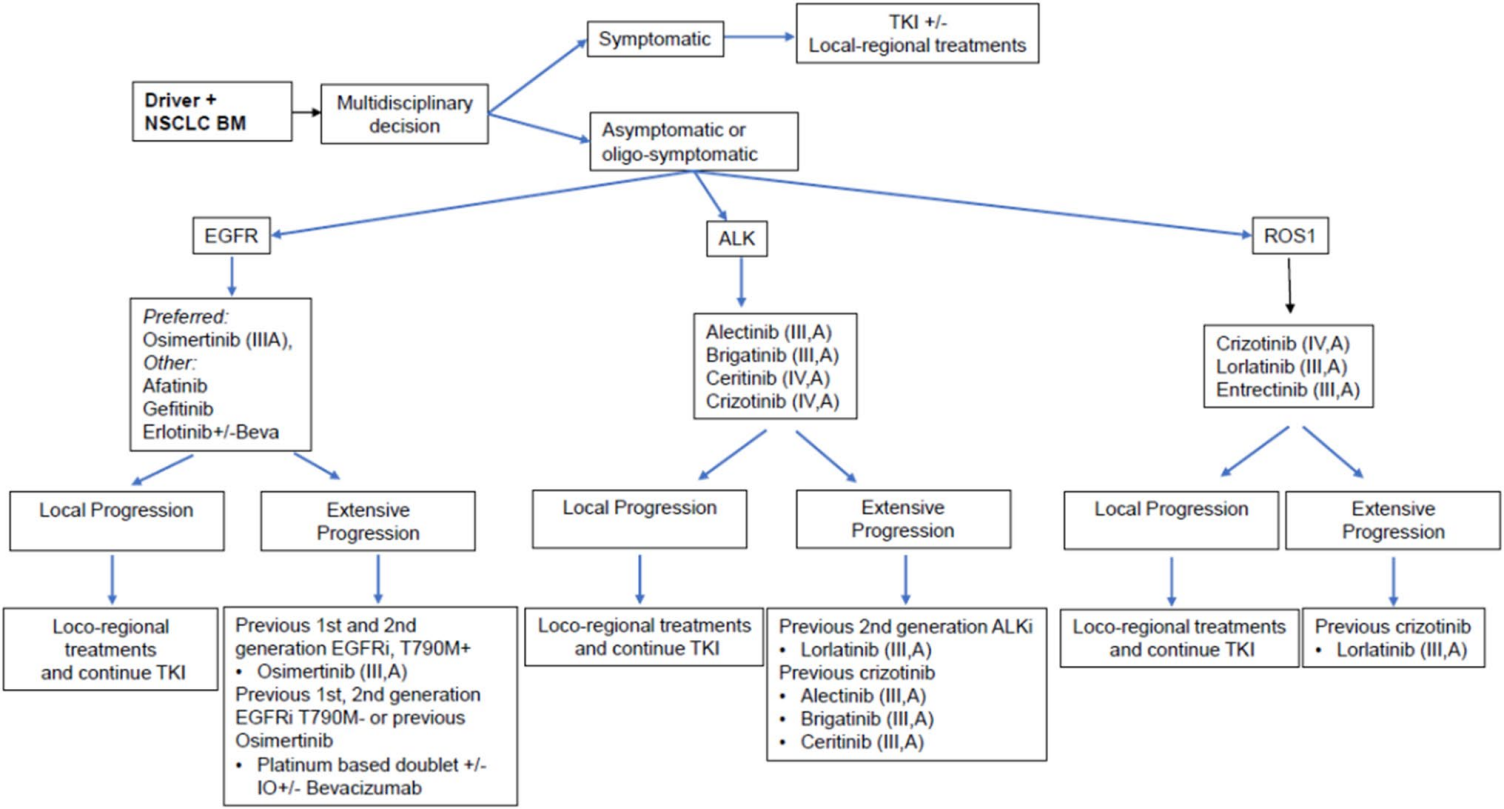
NSLC BM algorithm

NSCLC patients with EGFR mutations have a higher risk of developing BMs, ranging between 24% at diagnosis to more than 45% at 3 years post-diagnosis [31]. Multiple studies have demonstrated better activity in the CNS with 1st- and 2nd-generation TKIs compared to cytotoxic chemotherapy [32], although in general, the efficacy of 1st- and 2nd-generation TKIs is limited at the brain level mainly due to a modest penetration of the BBB. A retrospective study of 306 NSCLC patients compared the efficacy of 1st- and 2nd-generation EGFR TKIs showed no significant differences in the cumulative incidences of subsequent BM at 6, 12, and 24 months when comparing gefitinib vs erlotinib and afatinib (*p* = 0.80) [33]. Osimertinib, a 3rd-generation EGFR TKI, has a higher BBB penetration than 1st- and 2nd-generation EGFR TKIs. In previously treated EGFR^T790M^-positive advanced NSCLC, osimertinib was compared to pemetrexed/platinum combination in the randomized phase III AURA3 trial: CNS ORR was found to be higher in those patients receiving osimertinib vs pemetrexed/platinum (70% vs 31%, respectively) [34]. Similarly, a pooled analysis from two phase II studies that included patients with EGFR^T790M^-positive advanced NSCLC that progressed following treatment with an EGFR TKI showed a CNS relative risk of 54% and disease control rate of 92% [35]. In previously untreated EGFR-mutant advanced NSCLC, osimertinib was compared to gefitinib or erlotinib in the randomized phase III Flaura trial: 22% of patients that received osimertinib presented BM compared with 24% treated with erlotinib or gefitinib. The median CNS PFS was not reached with osimertinib and limited to 13.9 months with 1st-generation TKI (HR 0.48). CNS ORR was 91% and 68% in patients with ≥ 1 measurable CNS lesions and 66% and 43% in patients with measurable and/or non-measurable CNS lesions, respectively. The risk of CNS progression at 12 months was 8% with osimertinib and 24% with either gefitinib or erlotinib [36]. Based on the systemic and brain activity profiles, osimertinib can be considered the preferred 1st-line option for patients harboring EGFR mutations and BM (Fig. 3).

### ALK-rearranged NSCLC

*ALK*-positive NSCLC is characterized by a high incidence of BM (24–48%) [31]. In the 1st-line setting, 1st-generation *ALK* inhibitor (ALKi) crizotinib demonstrated higher intracranial disease control rate over chemotherapy. However, progression in the brain remained a significant clinical problem with crizotinib treatment [37, 38]. A 2nd-generation ALKi, ceritinib, significantly improved intracranial response rate (icRR) over chemotherapy (72.7% vs 27.3%, respectively). Nevertheless, due to the toxicity profile its use has not been commonly extended [39]. Alectinib and brigatinib are 2nd-generation ALKi that have shown a significant improvement in icRR (81–78% and 50–29%, respectively) and longer time to CNS progression over crizotinib (HR 0.16; 95% CI 0.10–0.28 vs HR 0.30; 95% CI 0.15–0.60; respectively) [40–42]. Similarly, upfront treatment with a 3rd-generation ALKi with broad range activity against *ALK*-resistant mutations achieved a significantly higher icRR and longer time to CNS progression compared to crizotinib (HR 0.07; 95% CI 0.03–0.17; 82% vs 23%, respectively) [43]. In patients progressing to frontline crizotinib, next-generation ALKi demonstrated promising CNS activity (icRRs ranging from 67 to 35%) [44–47]. In patients previously treated with at least one prior 2nd-generation ALKi and only one prior 2nd-generation ALKi, lorlatinib demonstrated an icRR of 56.1% and 66.7% and prolonged median intracranial duration of response of 12.4 months (95% CI 6.0–37.1) and 20.7 months (95% CI 4.1–37.1), respectively [48]. Data with other ALKi in the post-2nd-generation ALKi setting are still limited.

### ROS1-rearranged NSCLC

Up to 36% of patients with *ROS1*-positive NSCLCs have a BM at the diagnosis of metastatic disease [49]. In this NSCLC population, crizotinib was approved as the standard 1st-line treatment, however, suboptimal CNS penetration has been observed [50]. Next-generation ROS1 inhibitors (ROS1i) such as lorlatinib have demonstrated remarkable intracranial activity in both ROS1i-naive and crizotinib-pretreated patients (64% and 50%, respectively) [51]. Entrectinib, a multikinase inhibitor with activity against ROS1, demonstrated CNS activity in ROSi-naïve patients with an icRR of 55% (79.2% in patients with measurable disease) [52]. CNS efficacy data with other next-generation ROS1i are still limited.

## General recommendations for NSCLC BM

Figure 3.

- We recommend a baseline brain MRI for patients with oncogene-addicted NSCLC [IV, A]
- In patients with asymptomatic and multiple BMs and NSCLC with driver alterations for whom TKIs with high efficacy and brain penetrance are available, we consider it reasonable to use these targeted therapies as a 1st-line treatment and to delay the use of whole brain radiation therapy [II, A]
- Mildly symptomatic patients may still be considered for treatment with a TKI given the high icRR and the expected rapidity of response if close monitoring is ensured and endorsed by multidisciplinary discussion [IV, A]
- Patients with large volume, life-threatening, or highly symptomatic BM involvement should receive loco-regional treatments (either salvage stereotactic radiosurgery (SRS) or whole brain radiation therapy (WBRT)) based on the number and dimensions of the lesions, in addition to the appropriate systemic therapies [III, B]
- EGFR
  o Patients with BMs should be included in clinical trials whenever possible
  o At 1st-line setting, in EGFR TKI-naïve patients with asymptomatic or symptomatic multiple BMs, it is advisable to start treatment with osimertinib [III, A]
  o In EGFR^T790M^-positive patients progressing to 1st- or 2nd-generation EGFRi, the 3rd-generation TKI osimertinib is recommended [III, A]
- ALK
  o BM patients should be included in clinical trials whenever possible
  o Crizotinib is not the preferred 1st-line agent for BM patients due to its low ability to penetrate the BBB [IV, A]
  o At 1st-line setting, the 2nd-generation ALKi alectinib [III,A], brigatinib [III, A], and ceritinib [IV, A] could be considered standard treatment; however, due to safety profiles, alectinib and brigatinib are the preferred options
  o In patients progressing to crizotinib in the CNS, a second-generation ALKi is recommended [III, A]
  o In patients who progress to a 2nd-generation ALKi (alectinib or ceritinib as the first ALKi; or after crizotinib and at least one other ALKi), lorlatinib is recommended [III, A]
- ROS1
  o BM patients should be included in clinical trials wherever possible
  o Crizotinib is the only approved 1st-line agent for patients with ROS1-positive disease [IV, A]

## Management of BM in elderly patients

Elderly cancer patients require specific management (Fig. 3). Aging modifies functional reserve, and, together with comorbidity and geriatric syndromes, situates this population in a vulnerable position for the toxicity of treatments. Therapeutic decisions in elderly cancer patients should be based on geriatric assessment and at a level I of evidence [53–56]. Individualized treatment decisions for elderly cancer patients affected by BMs are a priority. An aggressive approach could be deleterious. To avoid toxicity, we must identify functional reserve, frailty, and ability to respond. Furthermore, stress must be managed properly in a population with a limited life expectancy because of aging itself. Elderly patients are often treated differently than younger individuals due to concerns regarding tolerance and survival [56]. Studies have focused on patients with 1–3 metastases. Patients with 1–2 BMs have a better prognosis than those with multiple lesions and appear to benefit from more aggressive approaches than WBRT alone. However, the cut-off age for the definition of elderly patients is 65 years, an age that nowadays is not considered the definition of an aged person [57]. Prognostic factors and treatment criteria in this patient group (≥ 65 years and 1 lesion) are performance status, histology of primary tumor, and time between diagnosis and BM detection [57, 58]. There is a lack of evidence for specific management of elderly cancer patients with multiple BMs [59].

## General principles for surveillance and monitoring of BM

Based on contemporary data, median survival of BM patients exceeds 6 months for all major cancer types and ranges from 8 to 16 months depending on the primary tumor [60]. Up to 50% of surviving patients with BMs will develop new lesions or progression of previously treated lesions within 6–12 months of initial therapy. Recurrent disease may be amenable to treatment with SRS, surgery, or WBRT, depending on the overall condition of the patient and the extent and location of the disease. In limited BM and in multiple BMs, we recommend a brain MRI every 2 months for those treated with SRS alone and every 3 months for the rest for the first 2 years and then every 4–6 months indefinitely. Imaging to evaluate emergent signs and symptoms is appropriate at any time [3] [IIA].
